# Backache Worse from Noise

**Published:** 1887-10

**Authors:** C. S. Durand

**Affiliations:** Sedalia, Mo.


### BACKACHE WORSE FROM NOISE.

Editors H. P.:—The following symptom in a case now under my care I am not able to find in the proving of any drug. Pain in the sacrum of a dull, aching character, aggravated by noise. Not by all noises, however ; thunder or the firing of cannon does not affect her, while she suffers severely from a shrill noise though it is not loud. She mentions the crowing of cocks, the whistle of a distant locomotive, and the clash of switching freight trains as especially distressing. Sometimes when lying awake at night with no pain at all it is immediately induced by any of the last-mentioned sounds.

The case is improving quite rapidly, having had three doses of Rhus and three of Bry. in high potencies since April 25th. But the aggravation from noise still persists. This is the only peculiar or characteristic feature of the case which I have yet discovered, so it would be of no interest to report the case in full.

If any of the readers of The Homœopathic Physician can throw light on this case it will greatly oblige both a young doctor and an old sufferer.

C. S. Durand, M. D., Sedalia, Mo.

